# Identification of new IS*711 *insertion sites in *Brucella abortus *field isolates

**DOI:** 10.1186/1471-2180-11-176

**Published:** 2011-08-03

**Authors:** Marcos Mancilla, Marcos Ulloa, Ignacio López-Goñi, Ignacio Moriyón, Ana María Zárraga

**Affiliations:** 1 Instituto de Bioquímica, Facultad de Ciencias, Universidad Austral de Chile, casilla 567, Valdivia, Chile; 2 Instituto de Salud Tropical (IST) and Departamento de Microbiología y Parasitología, Universidad de Navarra, c/Irunlarrea 1, 31008, Pamplona, Spain

## Abstract

**Background:**

Brucellosis is a zoonosis caused by *Brucella *spp., a group of highly homogeneous bacteria. The insertion sequence IS*711 *is characteristic of these bacteria, and occurs in variable numbers and positions, but always constant within a given species. This species-associated polymorphism is used in molecular typing and identification. Field isolates of *B. abortus*, the most common species infecting cattle, typically carry seven IS*711 *copies (one truncated). Thus far, IS*711 *transposition has only been shown *in vitro *and only for *B. ovis *and *B. pinnipedialis*, two species carrying a high number of IS*711 *copies, but never in other *Brucella *species, neither *in vitro *nor in field strains.

**Results:**

We found several *B. abortus *strains isolated from milk and aborted fetuses that carried additional IS*711 *copies in two hitherto undescribed insertion sites: one in an intergenic region near to the 3' end of a putative lactate permease gene and the other interrupting the sequence of a *marR *transcriptional regulator gene. Interestingly, the second type of insertion was identified in isolates obtained repeatedly from the same herd after successive brucellosis outbreaks, an observation that proves the stability and virulence of the new genotype under natural conditions. Sequence analyses revealed that the new copies probably resulted from the transposition of a single IS*711 *copy common to all *Brucella *species sequenced so far.

**Conclusions:**

Our results show that the replicative transposition of IS*711 *can occur under field conditions. Therefore, it represents an active mechanism for the emergence of genetic diversity in *B. abortus *thus contributing to intra-species genetic polymorphism.

## Background

*Brucella *is a genus of bacteria causing brucellosis, a zoonosis that affects a large variety of mammals and that is readily transmitted to humans. The genus includes several classical species that can be distinguished by their preferential host range, surface structure, biochemical and physiological features, and genetic markers. This classification is reflected in some degree of genetic polymorphism, one of the main sources of which is the copy number and distribution of IS*711 *(IS*6501*) [1,2]. *B. melitensis *and *B. suis *contain seven complete IS*711 *copies [3]. *B. abortus *carries six complete and one truncated IS*711 *copies [4], *B. ceti *and *B. pinnipedialis *more than 20 copies [5,6] and *B. ovis *38 copies [7]. IS*711 *is very stable: its mobility has been demonstrated only by using a "transposon trap" *in vitro *in *B. ovis *and *B. pinnipedialis*, but not in *B. melitensis *and *B. abortus *[3]. Based on this stability, polymorphism at the *alkB *locus [8] is used to differentiate *B. abortus *from *B. melitensis, B. ovis *and *B. suis *in the AMOS multiplex PCR assay [9].

IS*711 *stability is not only relevant for *Brucella *typification: its mobility is implicated in the generation of genetic diversity and speciation, as shown by the distribution of IS*711 *among the extant *Brucella *species. Here we report that IS*711 *transposition and the generation of the associated polymorphism takes place in *B. abortus *under natural conditions, when genetic drift should be limited by the selective pressure imposed by the host.

## Results and discussion

**Figure 1:**
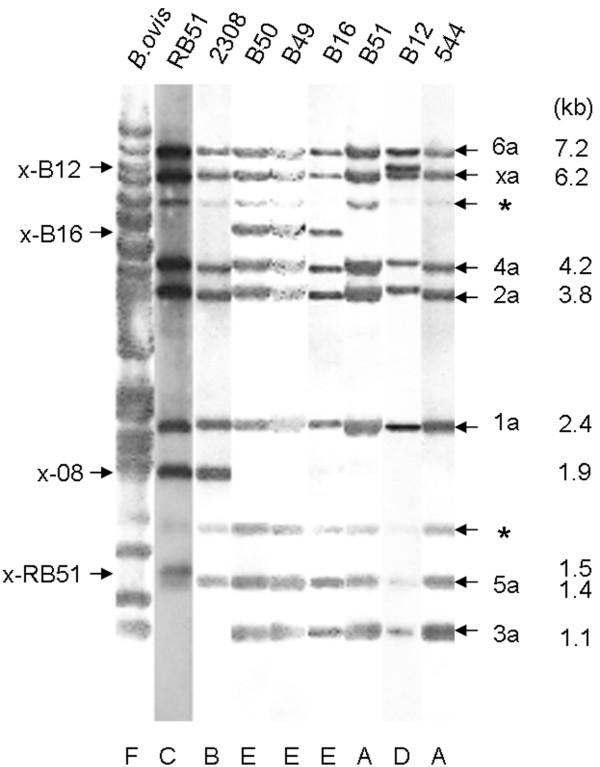
**Identification of new IS*711 *copies in *B. abortus *B12, B16, B49 and B50 by Southern blot**. The new IS*711 *copies found in field isolates and the additional IS*711 *present in 2308 and RB51 are indicated on the left. The IS*711*-nomenclature proposed by Ocampo-Sosa et al. (2008) and the fragment size are indicated on the right (note that x-08 fragment includes both the additional 2308 strain and 3a copies). The signals marked with an * correspond to IS other than IS7*11 *which show cross-hybridization. Capital letters at the bottom indicate the RFLP IS*711 Ava*I*-Cla*I profile (Table 1).

In a previous work with 46 *B. abortus *strains, it was found that two isolates (B12 and B16) displayed IS*711 *profiles that were different from that typical of *B. abortus *field strains [10]. This is confirmed here by the genetic profiling summarized in Table 1, and by the IS*711 *Southern blot presented in Figure 1. The latter shows that, while the reference strain *B. abortus *544 presented seven IS*711-*carrying fragments, isolates B12 (x-B12), and B16, B49 and B50 (x-B16) displayed an additional one. It is known that RB51, a lipopolysaccharide rough strain obtained from *B. abortus *2308 by multiple *in vitro *passages on antibiotic containing media, harbors eight copies plus an additional one that transposed into the lipopolysaccharide *wboA *gene [11]. Similarly, *B. abortus *2308, a strain isolated more than sixty years ago and extensively replicated in different laboratories carries eight IS*711 *copies [12,13]. However, the molecular weight of x-B12 and x-B16 fragments (6.6 and 5.5 kb, respectively) was different from those bearing the extra IS*711 *copies in 2308 (x-08, 1.9 kb that also includes the 3a copy) and RB51 (x-RB51, 1.5 kb) (Figure 1). Interestingly, whereas strain B51, which was isolated from the same sample as B12, displayed the genetic profile typical of *B. abortus*, strains B16, B49 and B50 showed an identical profile, even though they were from successive outbreaks in the same flock (Figure 1 and Table 1). These results show that it is possible to find *B. abortus *field isolates with different IS*711 *distributions.

**Table 1:** *Brucella *strains used

		Genetic profile by:		
			
Strain	Relevant features	RFLP IS*711 Ava*I*-Cla*I*^a^*	AMOS enhanced PCR *^b^*	Reference
*B. abortus *544	Reference strain of biovar 1	A	A	[24]
*B. abortus *2308	USDA challenge strain; biovar 1	B	B	[25]
*B. abortus *RB51	Vaccine rough derivative from 2308	C	B	[26]
*B. abortus *B51*^c^*	Biovar 1; milk isolate (Río Bueno, Chile; 2004)	A	A	This work
*B. abortus *B12*^c^*	Biovar 1; milk isolate (Río Bueno, Chile; 2004)	D	A	[10]
*B. abortus *B16*^d^*	Biovar 1; aborted fetus isolate (Osorno, Chile; 2002)	E	A	[10]
*B. abortus *B49*^d^*	Biovar 1; aborted fetus isolate (Osorno, Chile; 2000)	E	A	This work
*B. abortus *B50*^d^*	Biovar 1; aborted fetus isolate (Osorno, Chile; 2004)	E	A	This work
*B. ovis *23/290	*B. ovis *reference strain	F	C	[24]
*B. ceti *NCTC 12891^T^	*B. ceti *type strain	Np*^e^*	Np	[27]
*B. pinnipedialis *NCTC 12890^T^	*B. pinnipedialis *type strain	Np	Np	[27]
*B. abortus *2308 Nal^R^	Nalidixic acid resistant derivative of 2308 strain	Np	Np	[21]

*^a ^*IS profiles are shown in Figure 1.

*^b ^*A, *B. abortus *typical pattern; B, *B. abortus *2308 pattern; C, *B. ovis *typical pattern.

*^c ^*B12 and B51 were isolated from the same sample.

*^d ^*B16, B49 and B50 are strains isolated from different outbreaks in the same flock.

*^e ^*Np: Not performed

**Figure 2:**
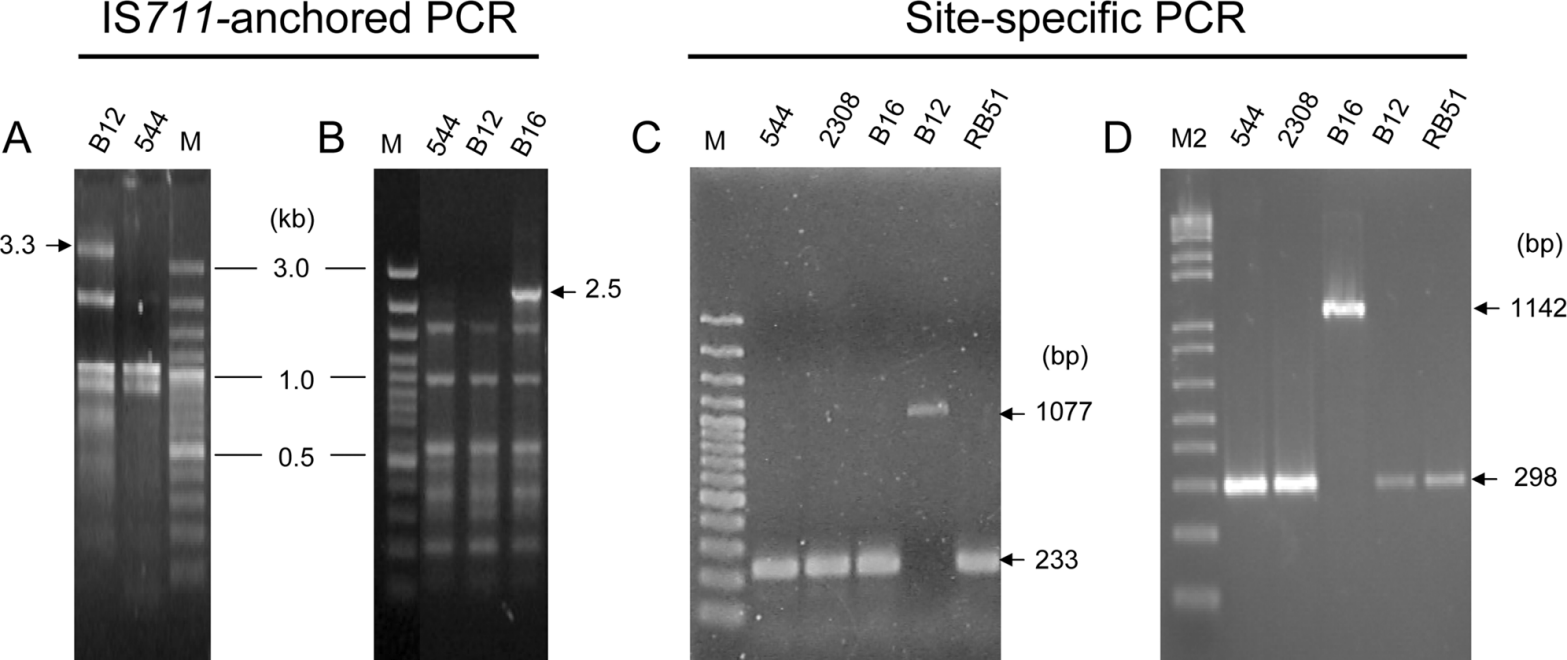
**PCR identification and characterization of new IS*711 *insertion sites in *B. abortus *B12 and B16 field isolates**. IS*711*-anchored PCR with: (A), primers IS711out-P5; or (B), RB51-P5. Site-specific PCR with: (C), primers BruAb1_0736F and BruAb1_0737R; or (D), forward and reverse primers of BruAb2_0461. For each lane, the number refers to the *B. abortus *strain used in the amplification. Arrows indicate specific PCR products generated from each strain. M, 1 kb DNA ladder (Fermentas); M2, 1 kb DNA ladder (Roche).

**Figure 3:**
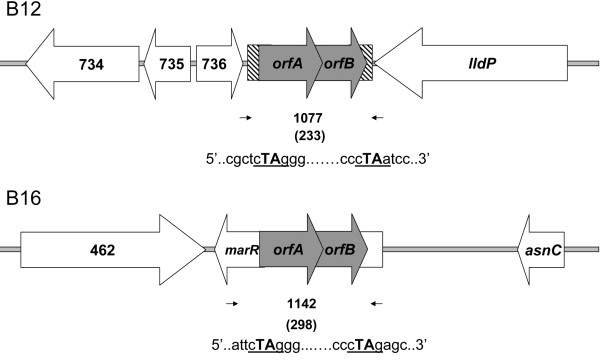
**Schematic representation of new IS*711 *loci found in *B. abortus *field isolates**. B12 (upper panel) and B16 and its related isolates (lower panel). The full-length 842 bp IS*711 *elements and their overlapping ORFs appear in grey. The Bru-RS1 element is shown as hatched box. The duplicated TA at the consensus YTAR site is shown below. Small black arrows represent the positions of site-specific primers. Numbers between primers indicate the molecular size of PCR products. The coordinates are based on the *B. abortus *9-941 annotation. ORFs BruAb1_0734, BruAb1_0735 and BruAb1_0736 encode hypothetical proteins; *lldP*, L-lactate permease (BruAb1_0737); BruAb2_462 encodes a putative D-amino acid oxidase family protein; *asnC*, transcriptional regulator AsnC family (BruAb2_0459).

We characterized the insertion sites in B12 and B16 (and B49 and B50) to ascertain whether they were new or already present in other brucellae. To this end, we carried out IS-anchored PCR using IS*711*-bound primers plus a decamer of %GC similar to that of the *Brucella *genome (Table 2). The resulting amplicons ranged from 0.2-3.3 kb (Figure 2A and 2B) with a similar distribution among strains, but with an additional PCR fragment for each B12 and B16 strains. Considering that those fragments may contain part of the additional IS copies plus their surrounding sequences, we cloned and sequenced the 3.3 kb and 2.5 kb DNA amplicons of B12 and B16, respectively, and designed flanking primers (Table 2) to confirm the position of the new IS copy. As predicted for the insertion of complete IS*711 *copies of 842 bp in length, specific PCR products of 1077 bp (B12) and 1142 bp (B16) were amplified (Figure 2C and 2D). We believe that an IS replicative transposition is the most plausible explanation for these results. In fact, the sequence analysis suggested that transposition had occurred by a canonical TA duplication at YTAR site (R, purine; Y, pirimidine). In strain B12, this site was in an intergenic region between a lactate permease gene (*lldP*) and BruAb1_0736 (hypothetical protein) (Figure 3, upper panel) corresponding to a 103 bp Bru-RS1 element, a palindromic repeat sequence that represents a putative insertion site for IS*711 *[14]. In contrast, the IS*711 *extra copy in B16, B49 and B50 was interrupting an ORF encoding a transcriptional regulator of the MarR family (BruAb2_0461, Figure 3 lower panel). Similarity searches showed that the B12 and B16 sites did not match with any of the IS*711 *loci previously reported for *B. abortus *or even with the novel IS*711 *sites recently described for *Brucella *marine mammal strains [6], although the B16 site was found in *B. ovis*. To confirm these findings and to investigate whether these sites were also present in the genomes (not available in databases) of the *Brucella *species carrying a high-copy number of IS*711*, we carried out PCR assays with *B. ovis, B. ceti *and *B. pinnipedialis *DNAs. For the B12-specific IS*711*, PCR amplifications with flanking primers yielded an IS-empty locus fragment (not shown). In contrast, the PCR amplifying the B16 fragment yielded the predicted 1142 bp fragment in *B. ovis *but not in *B. ceti *or *B. pinnipedialis *(Additional file 1).

**Table 2:** Primers used in this work

Name	Sequence (5'-3')	Reference
711d	CATATGATGGGACCAAACACCTAGGG	[19]
711u	CACAAGACTGCGTTGCCGACAGA	[19]
RB51	CCCCGGAAGATATGCTTCGATCC	[12]
IS711out	CAAGTTGAAACGCTATCGTCGC	This work
P5	CGGCCCCGGT	[20]
BruAb1_0736F	TTGGTTTCCTTGCGACAGAT	This work
BruAb1_0737R	AACCTTGCCTTTAGTTGCTCA	This work
BruAb2_0461F	ATCAGGCTTTGCTGGCAATC	This work
BruAb2_0461R	TCGTTTGCCATCTTGTTCAG	This work
*marR-*F1	GACGTGGTGGAGGAAACCTA	This work
*marR-*R2	ACTCGGCCAAACCTGATAA	This work
*marR-*F3	TTATCAGGTTTTGGCCGAGTCACATTGGAGTTGACCATCG	This work
*marR-*R4	CGCTTCGTGGTACGCTATTT	This work

**Figure 4:**
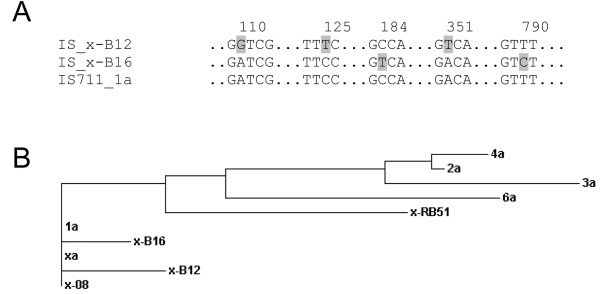
**Sequence analysis of IS*711 *copies found in *B. abortus *strains**. (A), Sequence alignment (IS*711*_1a is from *B. abortus *9-941). Single nucleotide polymorphisms are shadowed and numbered according to IS ORFs coordinates. (B), Clustering of full-length *B. abortus *IS*711 *copies found in *B. abortus *9-941 (note that truncated 5a copy was excluded), additional IS*711 *copy carried by *B. abortus *2308 (x-08) and *B. abortus *RB51 (x-RB51, accession no M94960), and the additional copies found in field isolates (x-B12, x-B16).

The x-B12 and x-B16 IS*711 *sequences were nearly identical to that of IS*711*_1a and depicted only changes in a few nucleotides (Figure 4A). On the basis of the high IS*711 *sequence similarity across sequenced *B. abortus *strains, we performed a cluster analysis between the IS*711 *copies of *B. abortus *9-941 and those additional ones found in 2308, RB51, B12 and B16 strains to get insight about their origin (Figure 4B). Although as expected, the analysis disclosed only low sequence dissimilarity, it suggested that the new copies might derive from IS*711*_1a. Since a previous work has shown that the IS*711*_xa in the *B. abortus alkB *locus and the IS*711_*x-08 in strain 2308 are identical to IS*711*_1a [3], the inclusion of IS*711*_x-B12 and IS*711*_x-B16 in the same cluster supports the hypothesis that IS*711*_1a is more active than other copies in the *B. abortus *genome and can transpose into new sites or even into sites shared with related species.

IS transposition can disrupt genes and produce negative polar effects, but also cause beneficial changes by remodeling genomes through long range recombination [15]. In the case of strain B12, it is uncertain whether the intergenic position of IS*711 *disturbs the expression of nearby genes. Most IS*711 *studied in detail (1a, 2a, 3a, 5a, 6a, xa and x-08) are also located within intergenic regions showing that transposition is mostly viable when occurring into neutral sites. However, the extra IS*711 *copy in B16, B49 and B50 interrupts a putative transcriptional regulator that is expressed during the late-logarithmic phase of growth in *B. melitensis *(BMEII0520) [16] and, interestingly, these strains did not show urease activity, a factor that has been proposed to favor *Brucella *gastrointestinal infections in mice [17]. We investigated whether the *marR *mutation was involved in the urease-negative phenotype by constructing a *B. abortus *2308 Δ*marR *mutant. This mutant displayed urease activity (not shown), suggesting that the absence of urease in B16, B49 and B50 is probably caused by mutation(s) in *ure *genes [17]. The fact that these urease negative *marR *mutant strains were repeatedly isolated from aborted fetuses for at least four years questions the relevance of this factor in placental colonization and abortion induction. Research is in progress to characterize the genetic background of this urease negative phenotype.

## Conclusions

In this report, we have provided evidence that IS*711 *polymorphism occurs in *B. abortus *field strains. The fact that such polymorphism can take place in sites shared with related species points out the relevance of a multiple-marker approach in molecular typing of *Brucella *species. In addition, our results suggest that the extra IS copies might originate from what seems to be the most active IS*711 *copy. Although the environmental signals involved in the activation of the transposase remain unknown, host-pathogen interactions may play a role. Further work is needed to elucidate if changes promoted by IS transposition are associated with virulence fluctuations in this pathogen.

## Methods

### Bacterial strains, growth conditions, plasmids and DNA manipulation

The *Brucella *strains studied are listed in Table 1 and the *E. coli *strains and plasmids used are in the Additional file 2. Bacteria were stored in tryptic soy broth (Becton Dickinson, Sparks, Md) with 20% glycerol at -70°C and, for routine use, grown on tryptic soy agar (when necessary under a 5% CO_2 _atmosphere) for 24-48 h at 37°C. Plasmids were obtained with Qiaprep (Qiagen, Hilden, Germany). PCR products and genomic DNA were purified with a QiaexII kit (Qiagen) or by standard protocols [18].

### Molecular typing techniques

AMOS PCR was carried out as described before [12]. For IS*711 *Southern blots, genomic DNA (1-2 μg) was digested with *Ava*I and *Cla*I (Fermentas Inc, Burlington, Canada) at 37°C overnight, the fragments resolved in 1.0% agarose at 15 mA for 10 h, blotted on nylon, fixed at 80°C for 30 min and probed with a biotin-labelled IS*711 *fragment obtained by PCR with primers 711u and 711d (Table 2). Hybridization was performed at 42°C for 2 h, and detected by chemiluminescence (KPL, Gaithersburg, MD) [19].

### Genome mapping of new IS*711 *insertion sites

For IS-anchored PCR, we adapted a protocol previously described [20]. IS*711*-bound primers RB51 and IS711out in combination with an arbitrary primer P5 (Table 2) were used to generate a pattern of PCR products specific for diverse IS positions. The reaction mixture contained 0.2 μM of RB51 or IS711out primers and P5 decamer, 5.0 μl of 10X enzyme buffer, 2 mM of MgCl_2_, 0.4 mM of dNTP, 1 U of Taq polymerase (Invitrogen) and 10 ng of genomic DNA. The amplification conditions were: 95°C for 5 min, followed by 30 cycles of denaturation at 95°C for 30 sec; annealing at 55°C for 30 sec; extension at 72°C for 2 min; final extension at 72°C for 5 min. Amplicons were electrophoresed in 1.5% agarose in 20 mM Tris, 20 mM acetic acid, 1 mM EDTA, and detected with ethidium bromide.

### Cloning and sequence analysis

Specific IS-anchored and flanking PCR products purified from gels were cloned into the pCR2.1 vector (Invitrogen) and sequenced by fluorescence-labeled dideoxynucleotide technology (Macrogen Inc, Seoul, South Korea). Sequences were analyzed by BLASTN (http://www.ncbi.nlm.nih.gov/). Comparison of the IS*711 *sequences in the *B. abortus *9-941 genome (accession numbers AE017223 and AE017224) [4] and the new IS*711 *was performed with ClustalW2 (http://www.ebi.ac.uk/Tools/clustalw2). Sequences of new IS*711 *were deposited under GenBank accession numbers: JF345125 and JF345126.

### Construction of B. abortus 2308 Δ*marR *mutant

A *B. abortus *2308 Nal^R ^Δ*marR *non polar mutant was constructed by allelic exchange [21] with primers designed on the sequence of *marR *(BAB2_0468, the *marR *homologous). Briefly, two fragments generated with primer pairs *marR-*F1, R2 and *marR-*F3, R4 (Table 2) were ligated by overlapping PCR and the resulting fragment (containing a Δ*marR *lacking the nucleotides corresponding to amino acids 13-120) was cloned into pCR2.1 to produce plasmid pMM19 (Additional file 2). The *Bam*HI-*Not*I fragment of pMM19 was subcloned into plasmid pJQK [22] to generate the pMM21 suicide vector (Additional file 2), which was transferred to *B. abortus *2308 Nal^R ^by conjugation with a suitable *E. coli *strain [23]. Nalidixic acid and sucrose resistant clones were screened by PCR, and tested for urease [17].

## Authors' contributions

MM conceived the study, participated in its design, accomplished computational analysis, and carried out molecular typing, mutagenesis and PCR assays. MU performed PCR assays and cloning procedures. ILG provided financial support and helped to draft the manuscript. IM and MM wrote the manuscript. AMZ participated in the design, coordination and financial support of the study and helped to draft the manuscript. All authors read and approved the final manuscript.

## Competing interests

The authors declare that they have no competing interests.

## Supplementary Material

Additional file 1 **PCR analysis for the presence of x-B16 fragment in *B. ovis, B. ceti *and *B. pinnipedialis***. Additional file 1 is a word file displaying a picture of PCR results. Click here for file

Additional file 2 ***E. coli *strains and plasmids**. Additional file 2 is a word file displaying a table with *E. coli *strains and plasmids used in this work. Click here for file
