# Supplementary material for: Identification of new IS711 insertion sites in Brucella abortus field isolates
Source: BMC Microbiol. 2011 Aug 3;11:176. doi: 10.1186/1471-2180-11-176 (PMC3163539; doi:10.1186/1471-2180-11-176)
Supplement: Additional file 1 — PCR analysis for the presence of x-B16 fragment in B. ovis, B. ceti and B. pinnipedialis. Additional file 1 is a word file displaying a picture of PCR results. [file 1471-2180-11-176-S1.DOC]

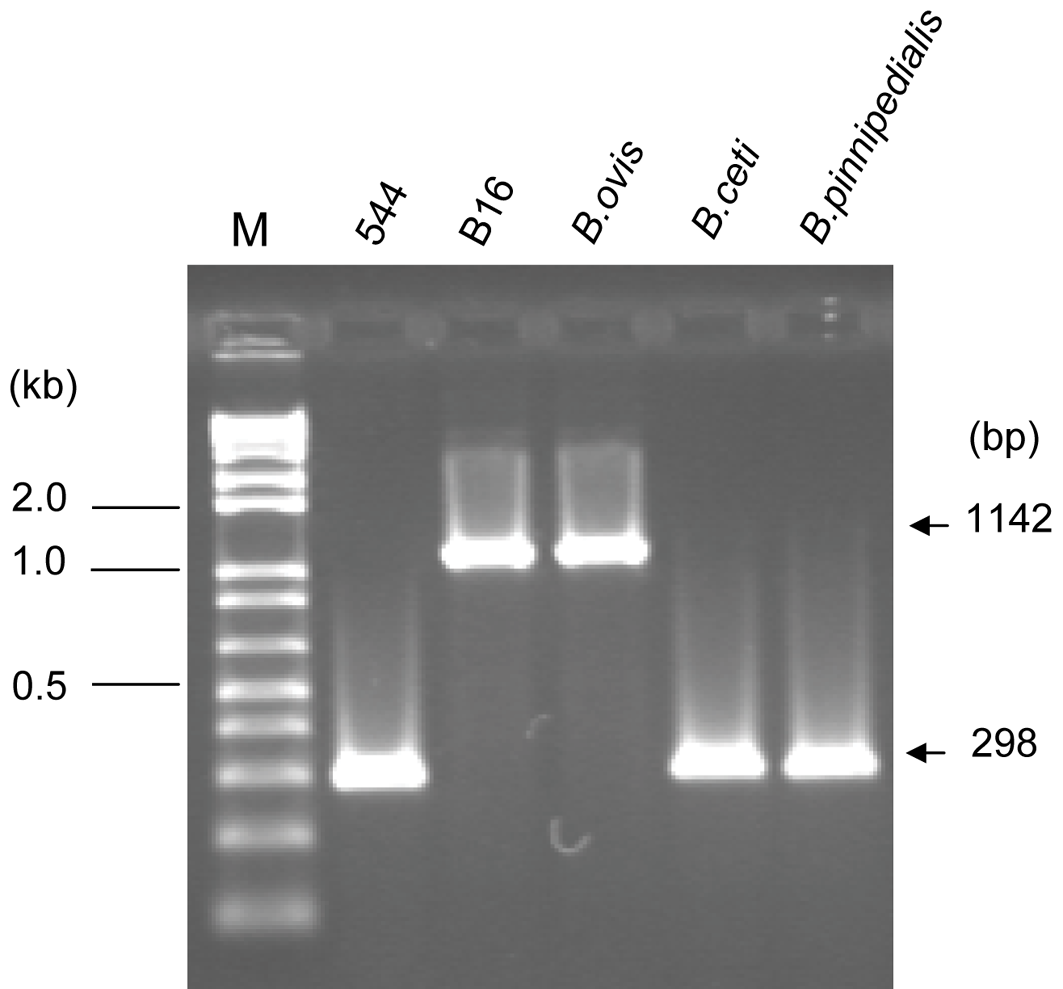


**Additional file 1. PCR analysis for the presence of x-B16 fragment in *B. ovis*, *B. ceti* and *B. pinnipedialis*.**

M, 1 kb DNA ladder (Roche).
