# Supplementary material for: Identification of new IS711 insertion sites in Brucella abortus field isolates
Source: BMC Microbiol. 2011 Aug 3;11:176. doi: 10.1186/1471-2180-11-176 (PMC3163539; doi:10.1186/1471-2180-11-176)
Supplement: Additional file 2 — E. coli strains and plasmids. Additional file 2 is a word file displaying a table with E. coli strains and plasmids used in this work. [file 1471-2180-11-176-S2.DOC]

**Additional file 2**: *E. coli* strains and plasmids used in this work.

| Strain/plasmid | Description | Source or reference |
| --- | --- | --- |
| S17-1λ*pir* | *E. coli* mating strain | [23] |
| TOP10 | *E. coli* cloning strain | Invitrogen |
| pCR2.1 | Cloning plasmid, kanamycin (Km) resistance | Invitrogen |
| pJQK | Derivative of plasmid pJQ200KS+, Km resistance | [22] |
| pX-B12 | pCR2.1 derivative containing a PCR fragment of approximately 2,5 kb derived from IS*711*-anchored PCR of B12 strain. | This work |
| pX-B16 | pCR2.1 derivative containing a PCR fragment of approximately 3,3 kb derived from IS*711*-anchored PCR of B16 strain. | This work |
| pB12 | pCR2.1 derivative containing a specific PCR fragment of 1077 bp from *B. abortus* B12 strain | This work |
| pB16 | pCR2.1 derivative containing a specific PCR fragment of 1142 bp from *B. abortus* B16 strain | This work |
| pMM19 | pCR2.1 derivative containing a deleted *marR*_Δ13-120_ copy | This work |
| pMM21 | Suicide vector, pJQK derivative containing the *Bam*HI-*Not*I fragment from pMM19 | This work |
